# Preparation and the assessed efficacy of oral baits for the vaccination of free-roaming dogs against rabies

**DOI:** 10.14202/vetworld.2022.1383-1390

**Published:** 2022-05-31

**Authors:** Naglaa I. Aly, Yasser F. Elnaker, Zeinab T. S. Salama, Mohamed S. Diab, Eman A. Saber, Sotohy A. Sotohy, Wael K. Elfeil, Mohamed H. Khodeir

**Affiliations:** 1 Department of Pet Animal Vaccine Research Veterinary Serum and Vaccine Research Institute, Agriculture Research Center, Ministry of Agriculture, Abassia, Cairo, Egypt; 2 Department of Infectious Diseases, Faculty of Veterinary Medicine, New Valley University, El-Kharga, Egypt; 3 Department of Animal Hygiene and Zoonoses, Faculty of Veterinary Medicine, New Valley University, El-Kharga, Egypt; 4 Department of Animal, Poultry and Environmental Hygiene, Faculty of Veterinary Medicine, Assiut University, Asyut, Egypt; 5 Department of Avian and Rabbit, Faculty of Veterinary Medicine, Suez Canal University, Ismailia, Egypt

**Keywords:** enzyme-linked immunosorbent assay, oral baits, potency, rabies, serum neutralization test

## Abstract

**Background and Aim:**

Rabies is considered a highly fatal zoonotic disease and many deaths in humans have been associated with dog bites. This study was designed to prepare an oral anti-rabies vaccine in the form of baits to eliminate the disease in free-roaming dogs and subsequently protect humans from dog bites.

**Materials and Methods:**

The Evelyn Rokintniki Abelseth (ERA) rabies virus strain was propagated in baby hamster kidney cell cultures and adjusted to the recommended dose for application. Four forms of oral baits were employed with the rabies vaccine, which was evaluated for safety, acceptability, and potency in different dog groups. Enzyme-Linked Immunosorbent Assay (ELISA) and the serum neutralization test (SNT) were used to determine the protective rabies antibody titer in the sera of vaccinated dogs.

**Results:**

According to the results, a dose of 3 mL of the ERA strain, containing a viral titer of 10^7.6^ TCID_50_/mL, induced a mean antibody titer of 25.6 by SNT, and the PI% was 75.7 by Block ELISA, providing a protective level of the rabies antibody in 100% of vaccinated dogs. All used baits were found to be safe, inducing no abnormal general post-vaccination signs (the signs are limited to mild fever, mild loss of appetite, and mild-to-moderate loss of energy for 24-36 h after vaccination).

**Conclusion:**

It was found that most of the accepted and highly potent bait types consisted of a mixture of wheat flour, vegetable oil, sodium alginate, corn starch, meat meal, cellulose gum, and water. This dog meal was covered with bran and edible wax to seal the bait cavity after inserting the vaccine sachet. This bait was able to induce a protective level of rabies antibodies in 100% of vaccinated dogs after receiving one bait/dog. Hence, such a bait could be recommended for use in the protection of free-roaming dogs and the elimination of the disease.

## Introduction

Rabies is a fatal viral zoonosis that affects the central nervous systems of warm-blooded mammals, including humans. It is caused by a negative single-strand ribonucleic acid virus belonging to the genus *Lyssavirus* of the family *Rhabdoviridae* [1]. Infection with this virus induces acute progressive encephalitis and results in approximately 60000 human fatalities annually, with 6-7 deaths occurring each day [2]. Most of these human rabies cases occur in Africa (36.4%) and Asia (59.6%). Most deaths occur in rural areas where access to health services, including post-exposure prophylaxis, is either limited or non-existent. More than 99% of all cases of human rabies infection occur through the bites of dogs infected with the virus [3-6]. Mass dog vaccination is an effective way of eliminating the disease, and the vaccination of ≥70% of the dogs in countries that are endemic to rabies could eliminate the infection in dogs and rapidly reduce the number of human cases [7-14]. However, it remains challenging to achieve that in Egypt because of the high proportion of free-roaming dogs that cannot be readily available for parenteral vaccination. Oral immunization of wildlife with live vaccines has been proven to be a powerful tool in controlling or eliminating rabies in multiple countries, such as those in North America and Europe [15,16]. Several types of recombinant or modified live attenuated vaccines were assessed for the oral rabies vaccination of dogs (OVD). The Evelyn Rokintniki Abelseth (ERA) strain is a cell-culture-adapted rabies virus derived from the Street-Alabama-Dufferin (SAD) and described as a live attenuated vaccine [17].

The ERA vaccine induces long-lasting immunity by intramuscular inoculation [18]. In the 1960s, Baer *et al*. [19] and Black and Lawson [20] demonstrated that foxes could be immunized against rabies through the oral administration of the live ERA virus. A vaccination program began in 1989 in the province of Ontario, Canada, for the control of terrestrial rabies in foxes. During this program, more than 13 million baits containing the live attenuated rabies ERA-baby hamster kidney-21 (ERA-BHK_21_) virus were distributed until the year 2004 [20,21]. The WHO Collaborating Center for Rabies Surveillance and Research in Tübingen, Germany, introduced the oral rabies vaccine virus SAD B19 as a possible candidate vaccine for the OVD as it is suitable for oral vaccination campaigns for carnivores (the red fox, *Vulpes vulpes*), dogs (*Canis familiaris*), cats (*Felis catus*), minks (*Mustela vision*), stonemartens (*Martes foina*), and domestic ferrets (*Mustela putorius furo*) against rabies [22-24]. A few trials are also underway to develop a vector-based vaccine for rabies containing either the Newcastle virus, Rift valley, adenovirus, or an adenovirus-like particle vaccine to potentiate worldwide efforts to control rabies [25-33]. The disease has been nearly eliminated in red foxes (*Vulpes* *vulpes*) in Ontario and in coyotes (*Canis* *latrans*) in south Texas using rabies vaccine oral baits [15,24,34-38].

This study aimed to develop an efficacious and attractive bait capable of delivering one effective dose of the oral rabies vaccine. The bait could be commercially manufactured at a low cost for large-scale use in the immunization of free-roaming dogs to eliminate rabies disease in free-roaming dogs and subsequently protect humans from dog bites.

## Materials and Methods

### Ethical approval

Animal care and experimental procedures were performed in compliance with guidelines established by the Institutional Animal Ethics Committee of the Veterinary Serum and Vaccine Research Institute, Abassia, Cairo, Ministry of Agriculture, Egypt (VSVRI), with approval no. VRA20119N1254.

### Study period and location

The study was conducted from January 2021 to December 2021 at the Veterinary Serum and Vaccine Research Institute, Abassia, Ministry of Agriculture, Cairo, Egypt (VSVRI).

### Preparation of the vaccine and the determination of the suitable dose

A cell-culture-adapted ERA rabies virus strain was kindly supplied in a lyophilized form at a titer of 10^4^ TCID_50_/mL by the WHO Collaborating Center for References and Research in Rabies (Pasteur, Paris-France). The virus was inoculated on a confluent sheet of the BHK_21_ cell line. When the maximum cytopathic effect (CPE) was established, the infected virus fluid was harvested, titrated, and tested for sterility; Viral titration was carried out using the micro-titer technique according to the WHO guidelines [39], and the viral titer was calculated according to the Reed and Muench method [40].

To determine the effective vaccinal dose, three different doses of the vaccine were tested (1, 2, and 3 mL/dog) by direct instillation in the buccal cavities of five dogs. After 28 days, the serum samples of the vaccinated animals were tested for the detection of induced rabies antibodies through the serum neutralizing test and enzyme-linked immunosorbent assay (ELISA).

### Vaccine preparation with different additives

#### Preparation of the rabies vaccine with a stabilizer

The stabilizer used was lactalbumin and sucrose consisting of 5% lactalbumin hydrolysate (LAH) and 10% sucrose in Hank’s Balanced Salt Solution with a pH of 7.2 mixed with an equal volume of the viral fluid [41].

#### Preparation of the rabies vaccine adjuvanted with 0.2% carbopol gel

Carbopol was supplied by Lubrizol Co. (Ohio, USA) as a white, fluffy powder. It was dissolved in hot water to prepare 0.5% aqueous stock solutions and sterilized by autoclaving at 121°C for 20 min, then stored at 4°C until further use according to the United States Pharmacopeial Convention guidelines [42]. An equal volume of the viral fluid was mixed with the aqueous solution of carbopol, and then neutralized with 20% sodium hydroxide [43]. Depending on the suitable vaccine dose in the results, the suitable dose was mixed with the stabilizer and 0.2% carbopol, and then dispersed into a polyethylene sterile sachet.

#### Preparation of an oral rabies vaccine with 1% carbopol gel

At first, potassium sorbate (as a preservative) was dissolved in distilled water at 40°C, and then 1% Carbopol was mixed with it using a magnetic stirrer at 160 × g for 30 min to homogenize. After cooling, a suitable dose of the vaccine was slowly added to the gel and homogenized to obtain a uniform gel. The pH of the gel was adjusted to 6 by adding triethanolamine to it [44].

#### Preparation of an oral rabies vaccine with sodium carboxymethyl cellulose (SCMC) gel

Potassium sorbate was dissolved in distilled water at 50°C. Then, an exact amount of SCMC (30%) was slowly added to it while mixing with a magnetic stirrer at 160 × g for 30 min to homogenize it fully. After cooling, a suitable dose of the vaccine was slowly added to uniformize the gel [44].

### Bait formation

Four types of baits were formed as follows:

Type 1: Flavor-coated sachets formed from Corn Syrup, Glycerin, Gelatin, Sucrose, Sodium Alginate, Corn Starch, Meat Meal, and Water.

The components of the formulation were admixed and then coated the sachet that contained the rabies vaccine by dipping the sachet in the formulation material. The coated sachet was then allowed to air dry.

Type 2: A fat wax mixture coated the sachet that contained the rabies vaccine by dipping the sachet into the mixture. The coated sachet was then dipped into cold water for rapid solidification of the coating and then stored in dry air.

Type 3: A hollow conical biscuit bait formed from maize flour with meat meal was filled with 3 mL of the rabies oral gel and closed with edible wax.

Type 4: A hollow cylinder bait formed from a mixture of wheat flour, vegetable oil, sodium alginate, corn starch, meat meal, cellulose gum, water, and dog food meal. After the insertion of the sachet, it was covered by bran and edible wax was poured on it to seal the bait cavity.

### Dogs

Eighty free-roaming, native breed dogs aged 6-12 months were collected from different areas and implemented in the safety and seroconversion assays. These dogs were found to be healthy and free from rabies antibodies as screened by the serum neutralization test (SNT). Dogs were held in individual cages under hygienic conditions, receiving balanced food and adequate water while being observed daily for approximately 6 months.

### Quality control tests of the prepared rabies vaccine

#### Sterility test

Samples of the prepared vaccine were inoculated in thioglycolate broth and nutrient agar and then incubated at 37°C for 7 days. Sabouraud’s agar, yeast, and mold broth were also inoculated and kept at room temperature (18-25°C) for 15 days. The results of sterility tests were negative for any bacterial and/or fungal growth before allowing the vaccine to proceed to the next step [39].

#### Safety test

Each of five healthy adult dogs was fed with five baits, each of which contained 3 mL of the attenuated rabies virus vaccine, and then kept under observation for 15 days. At the end of the observation period, the dogs should remain normal without any general reactions that can be attributed to the vaccine [39].

#### Potency test

Determine the efficacy of the vaccine without baits

This step was performed by directly instilling each of the prepared vaccine formulas mentioned before in the oral cavities of five dogs.

Determine the acceptability and efficacy of the prepared rabies vaccine oral baits

Forty dogs were divided into eight groups (five dogs per group). The dogs in each group were fed with one type of bait (one bait/dog with no booster doses). The last group, which was a control group, was made of dogs that remained unvaccinated to test the acceptability and the potency of the oral vaccine baits by monitoring the induced antibody titers in their sera on the 28^th^ day post-vaccination.

Serum samples obtained from the study dogs pre-, and 28 days post-vaccination were subjected to serological tests to estimate the induced antibody titers. The SNT was carried out using the micro-titer technique as described previously [45]. The antibody titer was expressed as the reciprocal of the highest serum dilution that neutralized and completely inhibited the CPE of 100 TCID_50_ of the used virus [46].

### Seroconversion

#### SNT

This test was carried out using the micro-titer technique described previously by Xuan *et al*. [45]. The antibody titer was expressed as the reciprocal of the highest serum dilution that neutralized and completely inhibited the CPE of 100 TCID_50_ of the used virus [46].

#### ELISA

The block ELISA method was employed to follow up on the induced rabies antibody titers in vaccinated dogs using an ELISA kit (Shenzhen Zhenrui Biotech Co, Ltd, Shenzhen, Chima). Positive and negative control sera were provided in the kit and the results were calculated according to the manufacturer’s instructions as follows:

Calculation method:


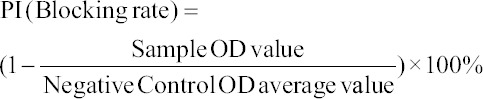


*The OD value of negative controls (N) was >0.5; meanwhile, a positive value blocking rate of >60% is equivalent to 0.5 IU/mL according to the standard curve.

## Results

### Sterility and safety

The experimental results revealed that all prepared vaccine formulas were free from foreign contaminants (aerobic and anaerobic bacteria and fungi) and safe in vaccinated dogs, where they remained healthy throughout the experimental period.

### Determination of the effective vaccinal dose

Table-1 shows that the preferable dose of the initial rabies virus with a titer of 10^7.6^ TCID_50 mL_ is 3 mL. This dose induced a mean antibody titer of 25.6 by SNT and a PI% of 75.7 by Block ELISA. It provided a protective level of rabies antibody in 100% of dogs compared to a dose of 2 mL that induced a mean SNT titer of 17.6 and a Block ELISA PI% of 58.6, with a protective level of rabies antibodies in 80% of dogs. However, a dose of 1 mL was unable to induce protective levels of rabies antibodies (4 by SNT and a PI% of 14.83 by Block ELISA) in all dogs in the group.

**Table 1:** Determination of the effective rabies oral vaccinal dose.

Used vaccine dose	Number of vaccinated dogs	SNT* titer	SNT mean	ELISA PI%**	ELISA PI% mean	Protection percentage
1 ml	5	2	Mean=4	9.70%	Mean=14.83	0%
		2		10.40%		
		4		15.85%		
		4		16.82%		
		8		21.4%		
2ml	5	8	Mean=17.6	24.3%	Mean=58.6	80%
		16		64.8%		
		16		62.1%		
		16		67.2%		
		32		74.6%		
3ml	5	32	Mean=25.6	78.3%	Mean=75.7	100%
		16		91.9%		
		16		68.3%		
		32		65.4%		
		32		74.6%		

* Mean SNT = the reciprocal of the final serum dilution which neutralized and inhibited the CPE of 100 TCID50 of rabies virus. (The protective SNT titer should not be less than 16).

** ELISA PI% is ˃60% equivalent to 0.5IU/ML

### Evaluation of the installation of different prepared rabies vaccine formulas in the buccal cavities of dogs

The results in Table-2 reveal that each of the prepared rabies vaccine formulas with stabilizers adjuvanted with 0.2% Carbopol and with gel with SCMC resulted in the production of a protective level of rabies antibodies in 100% of vaccinated dogs, with SNT mean titers of 28.8; 28.8, and 32 and mean ELISA PI% values of 67.2, 65.88, and 68.84, respectively. On the other hand, the vaccine prepared with 1% Carbopol Gel could not induce a sufficient level of RAB and showed a mean SNT titer of 4.4, and the ELISA PI% was 33.84%.

**Table 2:** Evaluation of the of the different prepared rabies vaccine formulae in the buccal cavity of dogs.

Used vaccine dose	Number of vaccinated dogs	SNT* titer	SNT mean	ELISA PI%**	ELISA PI% mean	Protection percentage
With stabilizer	5	32	Mean=28.8	71.3	Mean=67.2	100%
		32		69.4		
		32		65.8		
		16		62.1		
		32		67.4		
Adjuvated with 0.2% Carbopol	5	16	Mean=28.8	64.4	Mean=65.88	100%
		32		73.1		
		32		68.3		
		16		63.1		
		16		60.5		
Gel with 1% Carbopol	5	8	Mean=4.4	42.4	Mean=33.84	0%
		4		38.2		
		2		24.7		
		4		34.2		
		4		29.7		
Gel with SCMC	5	32	Mean=32	73.4	Mean=68.84	100%
		64		76.7		
		32		65.8		
		16		64.9		
		16		63.4		

* Mean SNT = the reciprocal of the final serum dilution which neutralized and inhibited the CPE of 100 TCID50 of rabies virus. (The protective SNT titer should not be less than 16).

** ELISA PI% is ˃60% equivalent to 0.5IU/ML

### Acceptability and efficacy of the prepared rabies vaccine oral baits

As per Table-3, the acceptance rate of baits of type 3 and 4 was 100% while those of type 1 and type 2 were 30% and 40%, respectively. The protective level of rabies antibodies obtained with the vaccinal bait type 4 and the stabilizer was 100% in all vaccinated dogs with a mean SNT titer of 25.6 and ELISA PI% of 68.56. This was followed by the vaccinal bait type 4 that contained the vaccine adjuvant Carbopol (0.2%) and brought about a protective level of the rabies antibody in 80% of vaccinated dogs, with a mean SNT titer of 30.4 and ELISA PI% of 66.58. On the other hand, the vaccinal bait type 1, which contained a stabilizer and was adjuvanted with 0.2% Carbopol, resulted in the production of protective levels of rabies antibodies in 20% and 40% of dogs with SNT titers and ELISA PI% values of 16 and 65.3 and 24 and 69.25, respectively. Furthermore, it was found that the administration of the vaccinal bait type 2 that contained a stabilizer and was adjuvanted with 0.2% Carbopol resulted in protective levels of rabies antibodies in 60% and 20% of dogs with SNT titers and ELISA PI% of 21.33 and 66.36 and 32 and 70.9, respectively. The vaccinal baits type 3, which contains the rabies vaccine with SCMC, resulted in protective levels of rabies antibodies in 20% of dogs with SNT titers and an ELISA PI% of 6.8 and 35.62, respectively.

**Table 3:** Determination of the acceptance and efficacy of the different type of rabies vaccine oral baits.

Type of baits	Used vaccine	Number of vaccinated dogs	Number of eaten baits (acceptance)	Acceptance rate	SNT* titer	SNT mean	ELISA PI%**	ELISA mean	Protection percentage
Type 1	With stabilizer	5	1	30%	16	-	65.3	-	20%
Type 1	Adjuvated with 0.2% Carbopol	5	2	30%	16 32	Mean=24	64.9 73.6	Mean=69.25	40%
Type 2	With stabilizer	5	3	40%	16 32 16	Mean=21.33	64.5 74.1 60.5	Mean=66.36	60%
Type 2	Adjuvated with 0.2% Carbopol	5	1	40%	32	-	70.9	-	20%
Type 3	Gel with SCMC	5	5	100%	8 2 16 4 4	Mean=6.8	46.7 21.6 63.8 24.6 21.4	Mean=35.62	20%
Type 4	With stabilizer	5	5	100%	32 16 16 32 32	Mean=25.6	74.6 62.1 64.2 69.8 72.1	Mean=68.56	100%
Type 4	Adjuvated with 0.2% Carbopol	5	5	100%	32 64 32 8 16	Mean=30.4	75.4 81.9 74.6 36.4 64.6	Mean=66.58	80%
Unvaccinated control group		-	5		-	-	09.0	-	0%

Type 1: Flavor coated sachet. Type 2: Fat wax mixture coated the sachet. Type 3: A hallow conical biscuit. Type 4: Hallow cylinder baits.

* Mean SNT = the reciprocal of the final serum dilution which neutralized and inhibited the CPE of 100 TCID50 of rabies virus. (The protective SNT titer should not be less than 16).

** ELISA PI% is ˃60% equivalent to 0.5IU/ML

## Discussion

Globally, almost all human deaths due to rabies are caused by dog bites, and approximately 99.9% of reported cases occur in Asia and Africa [39]. The oral vaccination of dogs (OVD) offers a new promising approach to achieve a significant increase in the dog vaccination coverage (especially among free-roaming and poorly-monitored dogs), when applied either exclusively or in combination with parenteral vaccination to boost herd immunity and the likelihood of disease elimination [47,48]. The present work is a trial to produce a potent oral rabies baits vaccine for the protection of free-roaming dogs to eliminate this disease. As the ERA strain of the rabies virus was used to control rabies in foxes during the 1989-2009 period, it led to a sharp decrease in the rate of post-exposure treatments in humans [49-51] and was used as field trials for OVD in Turkey and the Philippines [23,52]. A study by Aylan and Vos *et al*. [23] clearly showed the innocuity of SAD B19 for all mammal and bird species that were tested, and, most importantly, its performance over 15 years in the field without incidents. In this study, the virus was propagated in the BHK_21_ cell line until it reached a titer of 10^7.6^ TCID_50_/mL. This titer is sufficient for vaccine preparation, as mentioned previously by Lawson *et al*. [53]; so, the suggested dose of the virus vaccine suspension should have a titer of at least 10^6.8^ TCID/mL at the time of manufacture. The effective dose of the vaccine was estimated by direct instillation of three different doses of the vaccine (1 mL, 2 mL, and 3 mL) in the buccal cavities of the dogs (5 dogs/dose), with an initial titer of 10^7.6^ TCID_50_/mL. Rabies virus-neutralizing antibodies (RVNA) were estimated after 28 days in the sera of the vaccinated dogs, which revealed that the 3-mL dose produced a protective level of RVNA in 100% of the dogs, while the 2-mL dose produced a protective level of RVNA in 75% of dogs and a 1-mL dose did not produce a protective level in the sera of vaccinated dogs. Results of the SNT were recorded as the reciprocal of serum dilutions. Results of >16 were considered to be RVNA-positive [54] and, in ELISA, a test-positive value or a blocking rate (PI%) of >60% was equivalent to 0.5 IU/mL. As was reported in a recent study on foxes, it was shown that all vaccinated animals that developed titers of >0.5 IU/mL survived the challenge, whereas all vaccinated foxes with titers of <0.5 IU/mL succumbed to rabies [24]. Relatively high doses of the vaccine are needed to successfully immunize dogs (*Canis lupus familiaris*) by the oral route than foxes as discussed in previous studies [23,55]. Different additives were added to the vaccine as stabilizers (5% LAH and 10% sucrose) for prolonging vaccine stability and 0.2% Carbopol gel to increase the mucoadhesive properties of the vaccine to the mucosa of the buccal cavity. Furthermore, to facilitate the steps of vaccine production, an oral gel vaccine formed from 1% Carbopol and from 30% SCMC was prepared for direct filling into the baits without the need for a sachet. These two polymers are water-soluble and have mucoadhesive properties that are useful in pharmaceutical industries [56]. For the oral vaccines, the OIE recommends two efficacy studies, one involving direct vaccine instillation into the oral cavity and the other including bait delivery of the vaccine (vaccine in-bait efficacy) [57]. In this study, a satisfactory result was obtained through the application of direct vaccine instillation with different additives in the buccal cavities of the vaccinated dogs. The vaccine induced a protective level of serum neutralizing antibodies in dogs, except in the group of dogs that were vaccinated with the oral rabies vaccine containing 1% Carbopol gel, despite the formula that showed a higher viscosity and mucoadhesion properties due to its higher Carbopol content. Here the exhibited level of the antibodies was lower than the protective level, which may be attributed to the pH of the formula that affected the viability of the virus as shown in Table-2. The efficacy of the vaccine in baits was affected by the acceptability as in type one of baits with only three dogs out of ten accepting it. Four dogs out of ten in type two accepted it, while all dogs accepted types three and four and ate the baits within minutes. Hence, the percentage of dogs that produced protective levels of rabies antibodies differed according to the type of bait, to the extent that types one and two were acceptable for Raccoons and Coyotes [58] but seemed unacceptable for dogs. Interestingly, the high bait acceptability rate did not necessarily imply successful vaccination as shown in the case of the type three bait that induced a protective level of the rabies antibody in only 20% of vaccinated dogs, in spite of the high acceptability rate. The formula of the vaccine inside the bait was incorporated in the oral rabies gel with SCMC, which led to the rapid swallowing of the bait and, probably, the rapid inactivation or degradation of the rabies vaccine by the gastrointestinal tract. Optimally, the vaccine virus must be taken up in the oral cavity for the development of an immune response as reported by Bear *et al*. [21], which is supported by the findings from the study on the Köfte bait that was often swallowed completely without mastication, including the vaccine container used [59]. These results are shown in Table-3. All the dogs in the different groups remained healthy during the experiment that lasted for approximately 6 months without showing any signs of disease, as the viral strain used was a safe strain [8,60,61]. Perhaps a similar study needs to be conducted in the future with artificial infection in the vaccinated dogs to confirm the obtained results; however, due to the serious zoonotic potential of the rabies virus, such a study would require special precautions. Our results show that the prepared rabies vaccine’s oral baits protected free-roaming dogs against rabies and can help to eliminate the disease in Egypt.

## Conclusion

Formulated oral vaccines are easily administered to street dogs and can supplement the rabies control efforts of Egypt and other developing countries, but the main limitation is evaluating the vaccine application against challenge with field virus in the future work.

## Authors’ Contributions

NIA, WKE, YFE, ZTSS, MSD, and MHK: Conceptualization. NIA, MSD, EAS, and SAS: Methodology. WKE and MSD: Software. WKE, MSD, YFE, and NIA: Validation. NIA and MSD: Formal analysis. ZTSS: Investigation. MSD; WKE, and MHK: Resources. NIA, WKE, and MSD: Data curation. NIA and MSD: Drafted the manuscript. WKE: Reviewed and edited the manuscript. MSD: Visualization. MHK: Supervision. ZTSS: Project administration. MSD: Funding acquisition. All authors have read and approved the final manuscript.
